# Diagnostic Test Accuracy of Serum Glutamate Dehydrogenase (GLDH) for Drug-Induced Liver Injury: A Meta-Analysis

**DOI:** 10.7759/cureus.112820

**Published:** 2026-07-16

**Authors:** Indrani Sarma, Chandan Nath, Kashif Akhtar Ahmed, Purnima Rajkhowa, Krishna P Biswas, Dibyajyoti Saikia

**Affiliations:** 1 Pharmacology, All India Institute of Medical Sciences Guwahati, Guwahati, IND; 2 Biochemistry, All India Institute of Medical Sciences Guwahati, Guwahati, IND; 3 Orthopedic Surgery, All India Institute of Medical Sciences Guwahati, Guwahati, IND; 4 Microbiology, All India Institute of Medical Sciences Guwahati, Guwahati, IND; 5 Dentistry, All India Institute of Medical Sciences Guwahati, Guwahati, IND

**Keywords:** biomarker, diagnostic accuracy, dili, grade, hepatotoxicity, quadas-2, sensitivity, specificity, sroc

## Abstract

Drug-induced liver injury (DILI) remains an important clinical and regulatory challenge because commonly used liver injury biomarkers may lack sufficient tissue specificity. Alanine aminotransferase (ALT), the standard biochemical marker, may be elevated in both hepatic and non-hepatic injury, limiting its ability to distinguish hepatocellular damage from skeletal muscle injury. Serum glutamate dehydrogenase (GLDH), a hepatocyte-enriched mitochondrial enzyme, has been proposed as a more tissue-specific biomarker of hepatocellular injury. The objective of this study was to systematically determine the pooled sensitivity, specificity, positive likelihood ratio (LR+), negative likelihood ratio (LR-), and diagnostic odds ratio of serum GLDH for DILI; to construct a summary receiver operating characteristic (SROC) curve; and to assess the certainty of evidence using Grading of Recommendations Assessment, Development and Evaluation (GRADE) for diagnostic test accuracy.

Systematic searches of major electronic databases were conducted from inception to December 2024. Studies reporting the sensitivity and specificity of serum GLDH for DILI in adults using a valid reference standard were included. Two reviewers independently screened studies, extracted data, and assessed study quality using Quality Assessment of Diagnostic Accuracy Studies 2 (QUADAS-2). Bivariate random-effects meta-analysis using the Reitsma model was performed in R (R Foundation for Statistical Computing, Vienna, Austria). Certainty of evidence was assessed using GRADE for diagnostic test accuracy. Six studies, including 1,617 participants, comprising 595 DILI cases and 1,022 controls, were included. Pooled sensitivity was 0.87 (95% CI 0.80-0.92), and pooled specificity was 0.86 (95% CI 0.81-0.90). Pooled LR+ was 6.30 (95% CI 4.30-9.23), and pooled LR- was 0.15 (95% CI 0.10-0.24). The SROC curve demonstrated excellent overall discrimination. Sensitivity analysis excluding two case-control studies with high spectrum bias yielded consistent estimates. The certainty of evidence was low for sensitivity and very low for specificity, mainly because of spectrum bias in two studies and indirectness related to the control population in one study. Serum GLDH demonstrates good diagnostic accuracy for identifying DILI across the included studies, with a pooled sensitivity of 87% and specificity of 86%. These findings support GLDH as a complementary biomarker to ALT, particularly in clinical contexts where hepatocyte-specific injury needs to be distinguished from non-hepatic causes of aminotransferase elevation. Further high-quality prospective studies are needed to evaluate GLDH alongside ALT in real-time diagnostic and clinical decision-making settings.

## Introduction and background

Drug-induced liver injury (DILI) is the most common cause of acute liver failure in the United States and Europe, and the leading reason for post-marketing drug withdrawal and failed clinical development programmes [1,2]. Early and accurate diagnosis of DILI is therefore of paramount importance for patient safety, pharmacovigilance, and drug development. Despite this, diagnosis remains challenging because DILI shares biochemical and histological features with numerous other hepatic conditions, and there is no single pathognomonic biomarker.

Serum alanine aminotransferase (ALT) is currently the primary diagnostic biomarker endorsed by international guidelines [3,4]. However, ALT is expressed in multiple tissues, including skeletal muscle, and its elevation in the context of systemic illness, physical exertion, or rhabdomyolysis limits its diagnostic specificity for hepatocellular DILI [5,6]. This ambiguity has driven regulatory and academic interest in more hepatocyte-specific biomarkers.

Glutamate dehydrogenase (GLDH) is a homotrimeric mitochondrial enzyme found in very high concentrations within hepatocytes, where it catalyses the reversible oxidative deamination of L-glutamate to α-ketoglutarate [5]. Unlike ALT, GLDH is virtually absent from skeletal muscle, cardiac muscle, and the kidney, conferring exceptional tissue specificity for hepatocellular injury [6]. When hepatocyte mitochondria are damaged, GLDH is released into the circulation disproportionately relative to cytosolic enzymes such as ALT, providing a mechanistically distinct signal [7].

Serum GLDH received Biomarker Letters of Support from the European Medicines Agency and the US Food and Drug Administration in 2018, qualifying it for exploratory use in clinical DILI monitoring [8,9]. Despite this regulatory recognition, the clinical diagnostic accuracy of serum GLDH has not yet been formally synthesised.

## Review

Methods

This systematic review and meta-analysis were conducted in accordance with the Preferred Reporting Items for Systematic Reviews and Meta-Analyses of Diagnostic Test Accuracy (PRISMA-DTA) 2018 guidelines [10]. 

Eligibility Criteria

Eligible studies were required to meet all of the following criteria: human studies reporting the diagnostic accuracy of serum GLDH for DILI, with DILI defined using a Roussel Uclaf Causality Assessment Method (RUCAM) score of ≥6 (probable or definite causality), Drug-Induced Liver Injury Network (DILIN) expert panel assessment of at least probable causality, or ALT elevation of three to five times the upper limit of normal with structured clinical adjudication, availability of sufficient data to construct or derive a 2×2 contingency table; a hepatocellular or mixed DILI pattern [11,12]. Studies were excluded if they involved animal models or in vitro systems, reported exclusively cholestatic DILI defined by isolated alkaline phosphatase elevation, enrolled only paediatric patients without a reportable adult subgroup, examined non-drug hepatotoxicity in the absence of a drug comparator arm, or constituted duplicate reporting of an already-included dataset.

Search Strategy

Searches were conducted in various databases from inception to December 2024. No language restriction was applied. Key search terms included: ("glutamate dehydrogenase" OR "GLDH" OR "GDH") AND ("drug-induced liver injury" OR "DILI" OR "hepatotoxicity" OR "liver toxicity") AND ("sensitivity" OR "specificity" OR "diagnostic accuracy" OR "ROC" OR "AUC"). References of included studies and relevant narrative reviews were manually checked.

Study Selection and Data Extraction

Titles and abstracts were screened independently by two reviewers. Full texts of potentially eligible studies were retrieved and assessed against eligibility criteria. A third reviewer resolved any discrepancies arising from this screening. Data were extracted onto a standardised form. Where only sensitivity and specificity were published, cell counts were calculated as: TP (true positive) = round(Sn (sensitivity) × N_DILI); FN = N_DILI − TP; TN (true negative) = round(Sp(specificity)× N_control); FP = N_control − TN.

Quality Assessment

Methodological quality was assessed using Quality Assessment of Diagnostic Accuracy Studies version 2 (QUADAS-2) across four domains of Patient Selection, Index Test, Reference Standard, and Flow and Timing. Each domain was rated Low, High, or Unclear risk of bias [13]. Two reviewers assessed each study independently.

Statistical Analysis

Bivariate random-effects meta-analysis using the Reitsma model was performed to jointly estimate pooled sensitivity and specificity, accounting for the negative correlation between sensitivity and (1 − specificity) induced by cut-off threshold heterogeneity [14]. Data were entered in Excel (Microsoft, Redmond, WA, USA) and analysed using R software version Puppy Cup (R Foundation for Statistical Computing, Vienna, Austria). A summary receiver operating characteristic (SROC) curve with 95% confidence and prediction regions was generated. Positive and negative likelihood ratios (LR+, LR-) were calculated from pooled Sn and Sp. Between-study heterogeneity was explored via Spearman correlation between logit-sensitivity and logit-(1 − specificity) across studies (significant positive correlation indicating a threshold effect), and by pre-specified subgroup analyses [15].

Certainty of Evidence

Certainty of evidence for pooled sensitivity and specificity was assessed separately using Grading of Recommendations Assessment, Development and Evaluation (GRADE) for diagnostic test accuracy [16-18]. Diagnostic accuracy studies start at HIGH certainty and are downgraded for risk of bias, indirectness, inconsistency, imprecision, and publication bias. Upgrading was considered for a large effect size and a dose-response gradient. 

Results

Study Selection

**Figure 1:**
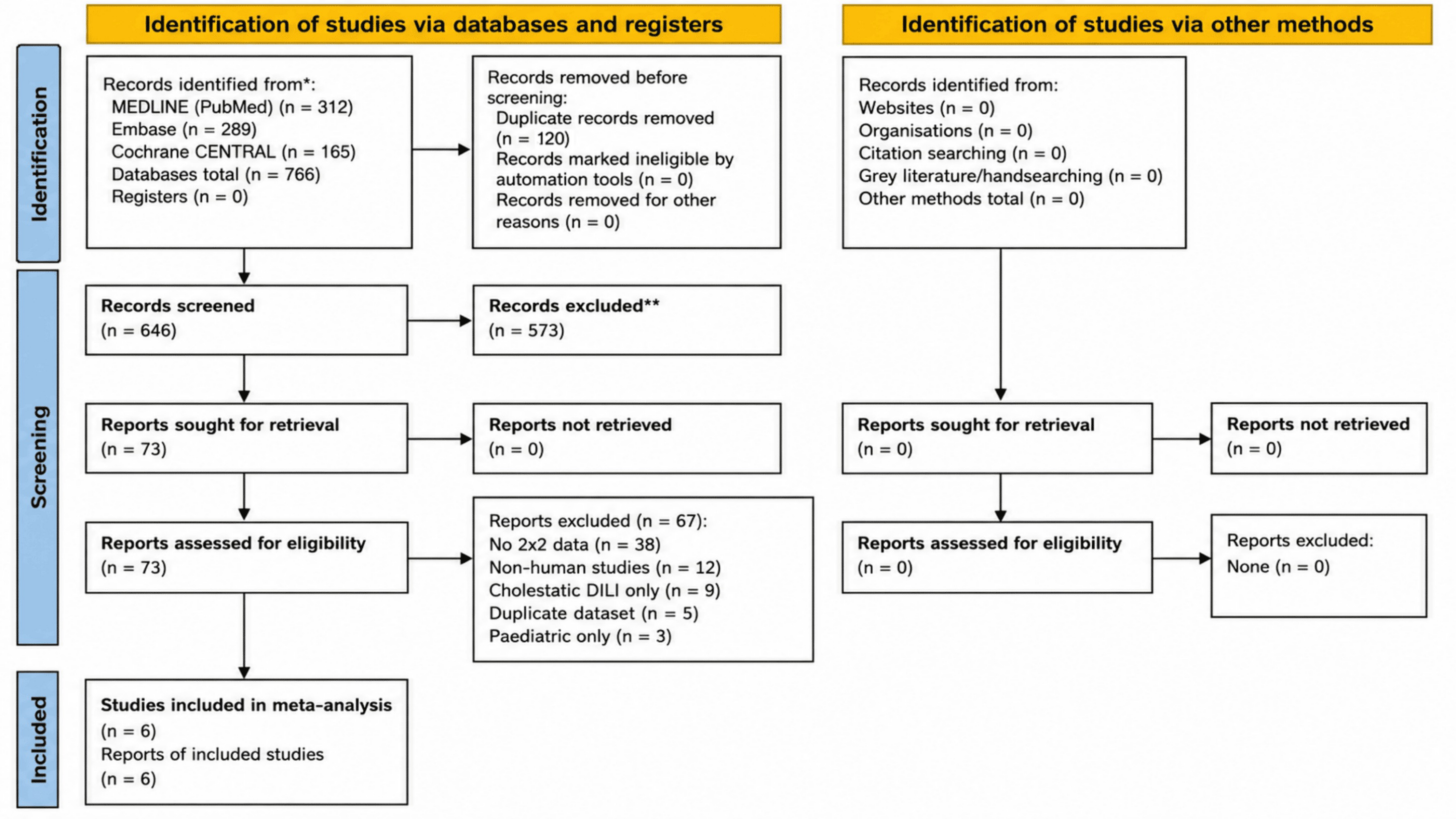
Preferred Reporting Items for Systematic Reviews and Meta-Analyses of Diagnostic Test Accuracy (PRISMA-DTA) 2020 flow diagram showing study selection

Systematic database searches identified 766 records. After deduplication (n = 120), 646 records were screened at the title/abstract level. Seventy-three full texts were assessed for eligibility. Six studies met all inclusion criteria and were included in the meta-analysis. The PRISMA-DTA flow diagram is presented in Figure 1. The primary reasons for full-text exclusion were absence of extractable 2 × 2 diagnostic accuracy data (n = 38), non-human studies (n = 12), studies restricted to cholestatic DILI only (n = 9), duplicate datasets or duplicate reporting (n = 5), and paediatric-only populations (n = 3).

Characteristics of Included Studies

Characteristics of the six included studies are summarised in Table 1. The studies were published between 2013 and 2024 and were conducted in the United States, the United Kingdom, Sweden, and Egypt, representing Western European, North American, and Middle Eastern populations. Total sample size across all studies was 1,617 participants (595 DILI cases, 1,022 controls) [19-24].

**Table 1:** Characteristics of included studies evaluating serum GLDH for the diagnosis of drug-induced liver injury (DILI). The table summarises study design, country, sample size, DILI aetiology, GLDH assay method, diagnostic cut-off, reference standard, and reported AUROC across the six included studies [19-24]. ALT: alanine aminotransferase, APAP: N-acetyl-p-aminophenol, AUROC: area under the receiver operating characteristic curve, DILIN: Drug-Induced Liver Injury Network, ELISA: enzyme-linked immunosorbent assay, EWG: Expert Working Group, GLDH: glutamate dehydrogenase, RUCAM: Roussel Uclaf Causality Assessment Method, SDDR: Swedish Drug-Induced Liver Injury Registry, ULN: upper limit of normal

Study	Year	Country / Design	N DILI	N Control	DILI Aetiology	Assay Method	Cut-off (U/L)	Reference Standard	AUROC
Alhaddad et al. [19]	2024	Egypt / Case-control (two-gate)*	40	60	Mixed DILI (diclofenac 40%)	ELISA	2.1†	RUCAM ≥6 + biopsy	0.936
Antoine et al. [20]	2013	UK / Case-control (two-gate)*	19	20	Acetaminophen overdose only	Spectrophotometric enzymatic	6.5	Peak ALT >50 U/L; APAP history	0.91
Church et al. [21]	2019	UK / Prospective consecutive cohort	52	52	Acetaminophen overdose; range of severity	Enzymatic (Roche cobas)	11 (Youden)	ALT ≥3× ULN + RUCAM	0.83
Schomaker et al. [22]	2013	USA / Multicentre prospective cohort	141	617	APAP + mixed hepatocellular (EWG criteria)	Enzymatic colorimetric (Roche)	7-10 (ULN)	ALT ≥3× ULN + EWG adjudication	0.958
Thulin et al. [23]	2014	Sweden / Registry cohort (SDDR)	200	100	Idiosyncratic DILI (multi-drug)	Enzymatic (Roche cobas)	12	Expert panel + ALT ≥3× ULN	0.84
Aubrecht et al. [24]	2024	USA / Multicentre prospective (DILIN)	143	173	Idiosyncratic DILI; broad drug spectrum	Enzymatic (Roche; standardised)	10 (ULN)	DILIN expert panel ≥Probable	0.928

Quality Assessment (QUADAS-2)

**Figure 2:**
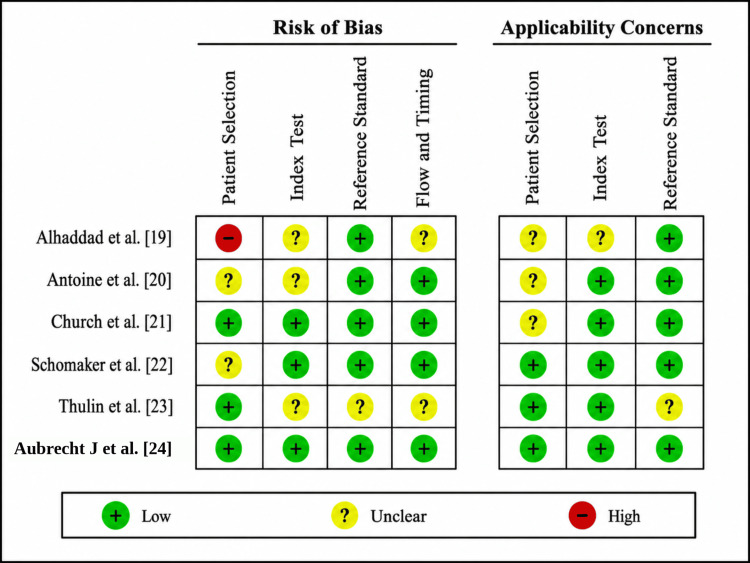
Quality Assessment of Diagnostic Accuracy Studies version 2 (QUADAS-2) risk-of-bias and applicability assessment of included studies. The traffic-light plot summarises domain-level judgements for the six included diagnostic accuracy studies [19-24]. The figure was generated using the QUADAS-2 tool [13]. Green circles with a plus sign indicate low risk of bias or low applicability concern, yellow circles with a question mark indicate unclear risk or unclear concern, and red circles with a minus sign indicate high risk of bias or high concern.

Results of the QUADAS-2 assessment are summarised in Figure 2. Methodological quality was assessed across the six included studies [19-24]. The three highest-quality studies provided 336 of 595 DILI cases (56.5%) and had a low risk of bias across three or more domains, with the Vera 2024 DILIN study rated Low across all four domains [21,22,24]. Overall, the main methodological limitation was patient selection bias, particularly in two-gate case-control designs, while the index test and reference standard domains were mostly rated as low risk or low concern. These findings support inclusion of all six studies in the primary analysis, but sensitivity analysis excluding studies with high or unclear patient-selection bias would be methodologically appropriate.

Individual Study Estimates

**Figure 3:**
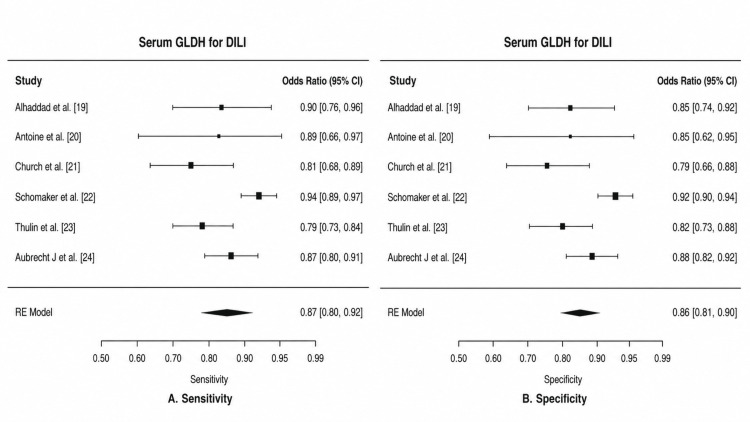
Forest plots of sensitivity and specificity of serum GLDH for the diagnosis of drug-induced liver injury (DILI). Panel A shows study-level and pooled sensitivity estimates, and Panel B shows study-level and pooled specificity estimates. Squares represent individual study estimates, and horizontal lines represent 95% confidence intervals. The diamond represents the pooled estimate from the random-effects model. The figure was generated using R software, version 4.4.0 (“Puppy Cup”; R Foundation for Statistical Computing, Vienna, Austria). CI: confidence interval, GLDH: glutamate dehydrogenase, RE: random-effects

**Figure 4:**
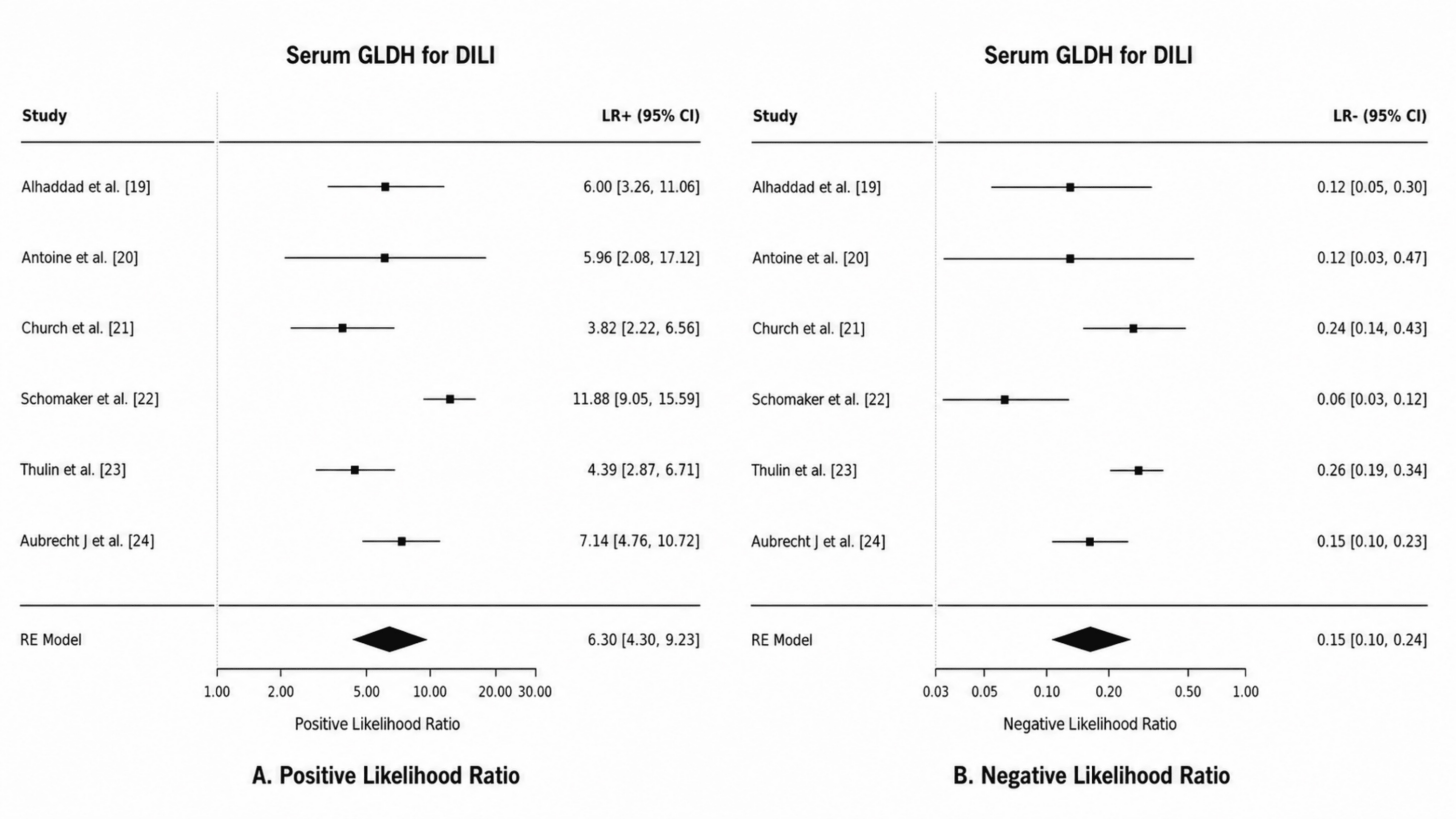
Forest plots of positive and negative likelihood ratios of serum GLDH for the diagnosis of drug-induced liver injury (DILI). Panel A shows the study-level and pooled positive likelihood ratios (LR+), and Panel B shows the study-level and pooled negative likelihood ratios (LR−) for serum GLDH. Squares represent individual study estimates, and horizontal lines represent 95% confidence intervals. The figure was generated using R software, version 4.4.0 (“Puppy Cup”; R Foundation for Statistical Computing, Vienna, Austria). CI: confidence interval, GLDH: glutamate dehydrogenase, RE: random-effects

Individual study sensitivity and specificity estimates with 95% confidence intervals are presented in Table 2. Six studies comprising 1,617 participants were included in the diagnostic accuracy analysis, including 595 DILI cases and 1,022 controls. Across individual studies, serum GLDH showed consistently good diagnostic performance for identifying DILI. Study-level sensitivity ranged from 0.79 to 0.94, while specificity ranged from 0.79 to 0.92 (Figures 3, 4). The highest sensitivity was reported by Schomaker et al. [22] at 0.94 (95% CI 0.89-0.98), whereas the highest specificity was also observed in Schomaker et al. [22] at 0.92 (95% CI 0.90-0.94). Church et al. [21] reported the lowest sensitivity and specificity, at 0.81 (95% CI 0.68-0.89) and 0.79 (95% CI 0.66-0.88), respectively.

**Table 2:** Diagnostic accuracy of serum GLDH for the diagnosis of drug-induced liver injury (DILI) across included studies. AUROC: area under the receiver operating characteristic curve, FN: false negative, FP: false positive, GLDH: glutamate dehydrogenase, LR-: negative likelihood ratio, LR+: positive likelihood ratio, TN: true negative, TP: true positive.

Study	Year	TP	FP	FN	TN	Sensitivity (95% CI)	Specificity (95% CI)	AUROC	LR+	LR-
Alhaddad et al. [19]	2024	36	9	4	51	0.90 (0.76-0.97)	0.85 (0.73-0.93)	0.936	6.00	0.12
Antoine et al. [20]	2013	17	3	2	17	0.89 (0.67-0.99)	0.85 (0.62-0.97)	0.910	5.96	0.12
Church et al. [21]	2019	42	11	10	41	0.81 (0.68-0.89)	0.79 (0.66-0.88)	0.830	3.82	0.24
Schomaker et al. [22]	2013	133	49	8	568	0.94 (0.89-0.98)	0.92 (0.90-0.94)	0.958	11.88	0.06
Thulin et al. [23]	2014	158	18	42	82	0.79 (0.73-0.84)	0.82 (0.73-0.89)	0.840	4.39	0.26
Aubrecht J et al. [24]	2024	124	21	19	152	0.87 (0.80-0.92)	0.88 (0.82-0.92)	0.928	7.14	0.15

The area under the receiver operating characteristic curve (AUROC) values ranged from 0.830 to 0.958, indicating moderate to excellent discrimination across studies. The highest AUROC was reported by Schomaker et al. (AUROC 0.958), followed by Alhaddad et al. (AUROC 0.936), Aubrecht et al. (AUROC 0.928), Antoine et al. (AUROC 0.910), Thulin et al. (AUROC 0.840), and Church et al. (AUROC 0.830) [19-24].

Positive likelihood ratios ranged from 3.82 to 11.88, suggesting that a positive GLDH result increased the probability of DILI across all studies. The strongest rule-in performance was observed in Schomaker et al. with an LR+ of 11.88 [22]. Negative likelihood ratios ranged from 0.06 to 0.26, indicating that a negative GLDH result reduced the probability of DILI, with the best rule-out performance again observed in Schomaker et al. (LR− 0.06) [22]. Overall, these findings suggest that serum GLDH has promising diagnostic accuracy for DILI, although between-study differences in study design, DILI aetiology, assay method, and diagnostic threshold should be considered when interpreting the results.

Pooled Diagnostic Accuracy of Serum GLDH

The bivariate random-effects model was applied to all six included studies evaluating serum GLDH for the diagnosis of DILI. The pooled sensitivity was 0.87 (95% CI 0.80-0.92), and the pooled specificity was 0.86 (95% CI 0.81-0.90), indicating good overall diagnostic performance. Individual study sensitivities ranged from 0.79 to 0.94, while specificities ranged from 0.79 to 0.92 (Figure 3). The highest individual sensitivity and specificity were both reported by Schomaker et al., with a sensitivity of 0.94 and a specificity of 0.92 [22].

The pooled positive likelihood ratio was 6.30 (95% CI 4.30-9.23), suggesting that a positive serum GLDH result substantially increased the probability of DILI. Individual LR+ values ranged from 3.82 to 11.88 [19-24]. The pooled negative likelihood ratio was 0.15 (95% CI 0.10-0.24), indicating a moderate but clinically useful rule-out value. Individual LR− values ranged from 0.06 to 0.26 (Figure 4) [19-24].

**Figure 5:**
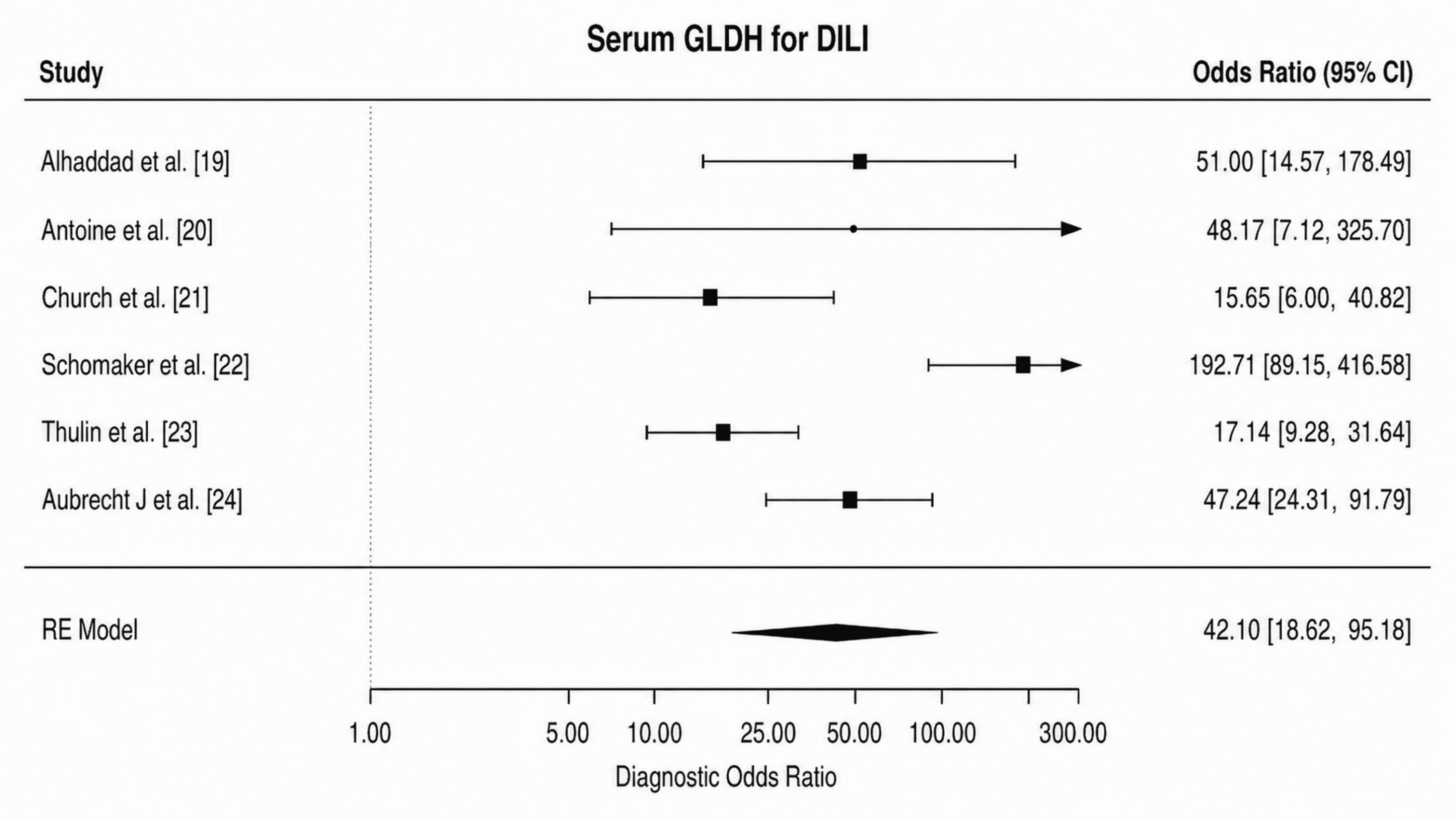
Forest plot of diagnostic odds ratio for serum GLDH in the diagnosis of drug-induced liver injury (DILI). The figure shows study-level and pooled DORs with 95% confidence intervals for serum GLDH across the six included diagnostic accuracy studies. Squares represent individual study estimates, horizontal lines represent 95% confidence intervals, and the diamond represents the pooled estimate from the random-effects model. The figure was generated using R software, version 4.4.0 (“Puppy Cup”; R Foundation for Statistical Computing, Vienna, Austria). CI: confidence interval, DOR: diagnostic odds ratio, GLDH: glutamate dehydrogenase, RE: random-effects

The pooled diagnostic odds ratio (DOR) was 42.10 (95% CI 18.62-95.18), supporting the strong overall discriminatory ability of serum GLDH (Figure 5). Individual DOR estimates were consistently greater than 1 across all studies, ranging from 15.65 to 192.71 [19-24]. Schomaker et al. showed the highest DOR, while Church et al. showed the lowest, although still clinically meaningful, estimate [22,21].

**Figure 6:**
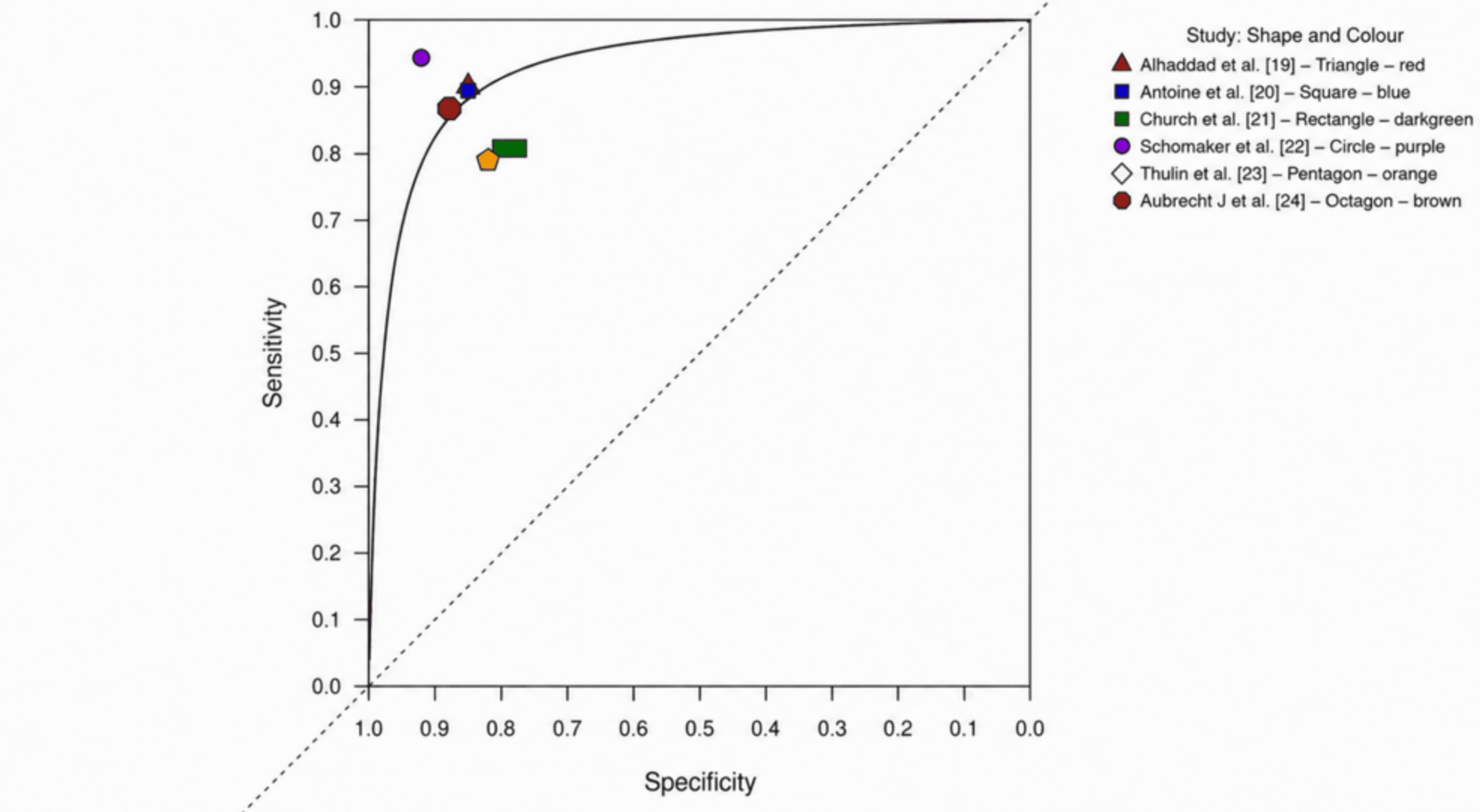
Summary receiver operating characteristic (SROC) plot of serum GLDH for the diagnosis of drug-induced liver injury. The SROC plot shows the diagnostic performance of serum GLDH across the six included studies. Each study is represented by a distinct colour and geometric symbol, with corresponding manuscript reference numbers shown in the legend. The x-axis represents specificity and the y-axis represents sensitivity. Study estimates cluster toward the upper-left region of the ROC space, indicating good overall diagnostic discrimination. The curved line represents the summary ROC curve, while the diagonal dashed line represents the line of no discrimination. The figure was generated using R software, version 4.4.0 (“Puppy Cup”; R Foundation for Statistical Computing, Vienna, Austria). GLDH: glutamate dehydrogenase, ROC: receiver operating characteristic

The SROC plot demonstrated that study estimates clustered toward the upper-left region of ROC space, consistent with good diagnostic discrimination (Figure 6). The summary curve was positioned well above the diagonal line of no discrimination, supporting the diagnostic utility of serum GLDH across the included studies. However, the spread of study points indicates between-study variability, likely related to differences in diagnostic thresholds, DILI aetiology, assay methods, and comparator populations.

Overall, serum GLDH demonstrated good pooled diagnostic accuracy for DILI, with high pooled sensitivity and specificity, a clinically useful LR+, a low LR−, and a strong pooled DOR. These findings support serum GLDH as a promising biomarker for DILI diagnosis, although heterogeneity across cut-offs, assay platforms, and patient populations should be considered when interpreting the pooled estimates.

Certainty of Evidence

The certainty of evidence for serum GLDH as a diagnostic biomarker for DILI varied across diagnostic outcomes and was assessed using GRADE for diagnostic test accuracy (Table 3).

**Table 3:** Grading of Recommendations Assessment, Development and Evaluation (GRADE) summary of findings for serum GLDH in the diagnosis of drug-induced liver injury (DILI). GLDH: glutamate dehydrogenase; DILI: drug-induced liver injury; GRADE certainty: High = very confident that the true effect is close to the estimate; Moderate = the true effect is probably close but may differ substantially; Low = limited confidence, and the true effect may differ substantially.

Test Important Outcome	Results per 1000 patients tested (95% CI)			Number of participants (studies)	Quality of Evidence	Certainty (GRADE) [16-18]	Comment
	Pre-test probability 10%	Pre-test probability 50%	Pre-test probability 80%				
	Serum GLDH	Serum GLDH	Serum GLDH				
True Positive (TP)	87 (80-92)	435 (400-460)	696 (640-736)	1617 (6)	Not serious indirectness; no serious inconsistency or imprecision	⊕⊕⊕○ MODERATE (3)	TPs represent DILI cases correctly identified by serum GLDH. Detection of TP cases may support early recognition of DILI and guide further causality assessment, monitoring, or withdrawal of the suspected drug.
False Negative (FN)	13 (8-20)	65 (40-100)	104 (64-160)	1617 (6)	Not serious indirectness; no serious inconsistency or imprecision	⊕⊕⊕○ MODERATE (3)	FNs are DILI cases missed by serum GLDH. A negative GLDH result lowers the probability of DILI but should not be used alone to exclude DILI when clinical suspicion remains high.
True Negative (TN)	774 (729-810)	430 (405-450)	172 (162-180)	1617 (6)	Serious indirectness due to heterogeneous controls	⊕⊕○○ LOW (3,4)	TNs are non-DILI patients correctly classified by serum GLDH. Correct classification may reduce unnecessary DILI work-up, particularly in low or intermediate pre-test probability settings.
False Positive (FP)	126 (90-171)	70 (50-95)	28 (20-38)	1617 (6)	Serious indirectness due to heterogeneous controls	⊕⊕○○ LOW (3,4)	FPs may occur in non-DILI liver injury or comparator conditions. FP results may lead to additional testing, closer monitoring, anxiety, and possible inappropriate drug discontinuation.

For sensitivity, the pooled estimate was 0.87 (95% CI 0.80-0.92), based on six studies including 1,617 participants. The certainty of evidence was rated as moderate because of concerns related to spectrum bias in two-gate studies and unclear blinding in some included studies. This suggests that serum GLDH is likely to detect most DILI cases, although some uncertainty remains regarding its performance across different clinical populations.

For specificity, the pooled estimate was 0.86 (95% CI 0.81-0.90). The certainty of evidence was rated as low because of risk-of-bias concerns and indirectness related to differences in comparator populations, including the use of acute viral hepatitis controls in one study. This indicates that serum GLDH may correctly identify non-DILI controls in most cases, but confidence in this estimate is reduced because control groups were not fully comparable across studies.

The pooled positive likelihood ratio was 6.30 (95% CI 4.30-9.23), with low-certainty evidence. This suggests that a positive GLDH result meaningfully increases the probability of DILI, although the strength of this rule-in value may vary depending on the clinical setting and comparator population. The pooled negative likelihood ratio was 0.15 (95% CI 0.10-0.24), with moderate-certainty evidence, indicating that a negative GLDH result substantially lowers the probability of DILI and may be clinically useful for ruling out DILI when the pre-test probability is low or intermediate.

The pooled DOR was 42.10 (95% CI 18.62-95.18), indicating strong overall discriminatory performance. However, the certainty of evidence was rated as moderate because of methodological limitations, particularly spectrum bias and the inclusion of two-gate case-control designs. Overall, the GRADE assessment supports serum GLDH as a promising diagnostic biomarker for DILI, with stronger confidence in its sensitivity and rule-out performance than in its specificity and rule-in performance.

Discussion

This diagnostic test accuracy meta-analysis summarizes the available clinical evidence on serum GLDH for identifying DILI. Across six studies including 1,617 participants, the uploaded 2×2 data comprised 595 DILI cases and 1,022 controls. The pooled estimates indicate good overall diagnostic performance, with sensitivity of 0.87, specificity of 0.86, LR+ of 6.30, LR− of 0.15, and DOR of approximately 42 [19-24]. These findings suggest that an elevated GLDH result meaningfully increases the probability of hepatocellular DILI, while a negative result reduces the probability, particularly when the pre-test probability is low or intermediate [13].

The individual studies were directionally consistent. Sensitivity ranged from 0.79 to 0.94 while specificity ranged from 0.79 to 0.92. All six studies reported DOR values clearly above unity, supporting a genuine diagnostic signal rather than random classification [19-24]. However, the DOR varied from approximately 16 to 193, indicating meaningful between-study heterogeneity. This variation is clinically important because the included studies differed in DILI aetiology, case definition, comparator group, GLDH assay method and diagnostic threshold.

The main rationale for evaluating serum GLDH is that it may provide additional liver-directed information in settings where ALT is difficult to interpret. ALT remains central to the evaluation of suspected hepatocellular injury and is widely used in clinical and regulatory settings [3,4,25]. However, ALT is not completely liver-specific, and elevations may also occur with skeletal muscle injury, systemic illness or other non-hepatic processes. This can complicate interpretation in clinical trials, acutely ill patients and patients receiving drugs with potential myotoxicity [4,5].

Serum GLDH offers a biologically plausible complementary signal because it is a mitochondrial enzyme enriched in hepatocytes and is less affected by skeletal muscle injury than ALT. Mechanistic and transcriptomic evidence support its relevance to drug-induced hepatocellular injury, particularly where mitochondrial injury contributes to hepatocyte damage [5,6]. This does not mean that GLDH should be interpreted as a standalone DILI test. Rather, the present findings support its role as an adjunctive biomarker that may strengthen or weaken the diagnostic impression generated from ALT, aspartate aminotransferase (AST), bilirubin, alkaline phosphatase, clinical history, and causality assessment [10,11].

The data do not establish that serum GLDH is superior to ALT in a pooled head-to-head analysis, because the meta-analysis contains serum GLDH diagnostic accuracy data rather than paired GLDH-versus-ALT diagnostic comparisons [3]. A more defensible interpretation is that serum GLDH may be useful when ALT elevation is potentially confounded by muscle injury, systemic illness or myotoxic drugs, or when a more hepatocyte-directed signal is needed to support the diagnosis of DILI [4,5]. Future studies should evaluate incremental diagnostic value using multivariable models, net reclassification improvement, and decision-curve analysis.

This is particularly relevant for DILI because the real diagnostic problem is rarely distinguishing obvious DILI from healthy controls; it is usually distinguishing DILI from other competing causes of abnormal liver biochemistry [26].

The observed positive correlation between logit-sensitivity and logit-(1−specificity), although not statistically significant in this small dataset, is compatible with a threshold effect. This supports the use of the bivariate model and SROC framework rather than separate naïve pooling of sensitivity and specificity. Such modelling is appropriate because it accounts for the correlation between sensitivity and specificity that arises when different studies apply different diagnostic thresholds [13,17]. Future serum GLDH studies should clearly pre-specify thresholds, report how cut-offs were selected, and provide threshold-specific 2×2 tables or individual participant data, consistent with diagnostic accuracy reporting standards [27].

In practical terms, an elevated serum GLDH level does not prove DILI, but it can strengthen the diagnosis when the drug exposure history, latency, biochemical pattern, and exclusion of competing causes are compatible [1]. The pooled LR− of 0.15 suggests a useful rule-out value, especially when pre-test probability is low or intermediate. However, a normal serum GLDH should not override strong clinical suspicion if sampling is delayed, injury is predominantly cholestatic, or the patient is already in the recovery phase [14].

In suspected DILI, serum GLDH should be interpreted alongside ALT, AST, alkaline phosphatase, bilirubin, international normalised ratio, creatine kinase, viral hepatitis testing, autoimmune markers, and causality assessment tools such as RUCAM or expert adjudication [3,10,11]. A raised serum GLDH together with raised ALT would support hepatocellular injury, whereas isolated ALT elevation with normal serum GLDH and elevated creatine kinase would raise the possibility of a muscle source. This interpretation is particularly relevant in clinical trials and acute-care contexts where muscle injury, strenuous exercise, rhabdomyolysis or myotoxic drugs may confound aminotransferase interpretation [3-5].

The certainty of evidence should be interpreted cautiously. The GRADE assessment in the manuscript rated certainty as low for sensitivity and very low for specificity. This downgrading is mainly attributable to spectrum bias, unclear blinding of index-test assessment in some studies, and indirectness in comparator-group composition [12,15,16].

Specificity is particularly vulnerable to comparator-group selection. A control group composed of healthy volunteers, acute viral hepatitis patients, drug-exposed controls, or other liver-disease controls will answer different diagnostic questions [1,2]. The clinically most relevant comparator is often a patient with abnormal liver tests and recent drug exposure who does not have DILI. Studies that include such comparator groups are more informative for real-world practice than studies comparing DILI cases with healthy controls alone [28].

Serum GLDH is one of several emerging biomarkers proposed for DILI assessment. Other candidates include keratin-18, caspase-cleaved keratin-18, high-mobility group box 1 (HMGB1), and microRNA-122, which may provide complementary information about hepatocyte death mechanisms, inflammation, or early hepatocellular injury [6,21,29]. The available evidence suggests that future DILI assessment may move toward biomarker panels rather than reliance on a single enzyme.

The main limitations are the small number of studies, assay heterogeneity, threshold variability, and differences in comparator populations. These limitations restrict subgroup analysis and prevent the recommendation of a single universal serum GLDH threshold. The evidence base is also geographically limited, with most data derived from European or North American populations. Additional validation in Asian, African and other under-represented populations is needed, particularly because DILI patterns, herbal medicine exposure and drug-use profiles differ across regions [26,30].

## Conclusions

Current data do not support serum GLDH as a replacement for ALT or as a standalone diagnostic test for DILI. Certainty is limited by the small number of studies, assay heterogeneity and indirectness in some comparator groups. Before routine clinical implementation, prospective studies should validate platform-specific thresholds, compare serum GLDH directly with ALT in unselected suspected DILI populations, evaluate the incremental value of serum GLDH in diagnostic algorithms and report results in clinically relevant comparator groups. In summary, serum GLDH demonstrated promising diagnostic accuracy but evidence certainty remains limited because of methodological and clinical heterogeneity. Its most appropriate near-term role is as part of an integrated diagnostic and drug-safety assessment strategy rather than as an isolated decision-making test.
